# A novel acupuncture technique at the Zusanli point based on virtual reality and EEG: a pilot study

**DOI:** 10.3389/fnins.2024.1269903

**Published:** 2024-05-09

**Authors:** Yongheng Zhang, Weicheng Hua, Ziqiu Zhou, Haibin Zhu, Jiawei Xiong, Jianbin Zhang, Duo Chen, Jiayang Guo

**Affiliations:** ^1^School of Artificial Intelligence and Information Technology, Nanjing University of Chinese Medicine, Nanjing, Jiangsu, China; ^2^The Second Affiliated Hospital of Nanjing University of Chinese Medicine, Nanjing, Jiangsu, China; ^3^National Institute for Data Science in Health and Medicine, Xiamen University, Xiamen, China; ^4^Department of Hematology, School of Medicine, Xiamen University, Xiamen, China

**Keywords:** TCM, acupuncture, VR, EEG, BCI

## Abstract

**Introduction:**

Acupuncture is a Traditional Chinese Medicine (TCM) method that achieves therapeutic effects through the interaction of neurotransmitters and neural regulation. It is generally carried out manually, making the related process expert-biased. Meanwhile, the neural stimulation effect of acupuncture is difficult to track objectively. In recent years, virtual reality (VR) in medicine has been on the fast lane to widespread use, especially in therapeutic stimulation. However, the use of related technologies in acupuncture has not been reported.

**Methods:**

In this work, a novel acupuncture stimulation technique using VR is proposed. To track the stimulation effect, the electroencephalogram (EEG) is used as the marker to validate brain activities under acupuncture.

**Results and discussion:**

After statistically analyzing the data of 24 subjects during acupuncture at the “Zusanli (ST36)” acupoint, it has been determined that Virtual Acupuncture (VA) has at least a 63.54% probability of inducing similar EEG activities as in Manual Acupuncture (MA). This work may provide a new solution for researchers and clinical practitioners using Brain-Computer Interface (BCI) in acupuncture.

## 1 Introduction

Acupuncture, a therapeutic method with a history of over 3,000 years, has been widely used in China and other Asian countries and was disseminated to Europe and America between the 16th and 19th centuries (Zhuang et al., 2013; Mallory et al., 2016). Patients undergoing acupuncture often experience various sensations such as numbness, soreness, pain, distention, heaviness, heat, or cold, and occasionally these feelings may spread or move along the meridians, a phenomenon known in Traditional Chinese Medicine (TCM) as “de Qi” or needle sensation (Hui et al., 2007; Lu et al., 2021; Zhang et al., 2022). From a neuroscientific perspective, acupuncture achieves its ultimate purpose through the complex interplay of the brain's functionally anticorrelated networks and neurotransmitters, and its effectiveness relies on psychophysical responses (Si et al., 2021; Wang et al., 2023).

The research of the “Zusanli” acupuncture point (ST36) is well-founded and highly beneficial, given its minimal side effects and substantial therapeutic potential (Lai et al., 2020). This acupuncture point plays a crucial role in regulating spleen and stomach functions, alleviating pain, balancing Qi and blood, enhancing immunity, and promoting metabolism (Zhou and Benharash, 2014; Lu et al., 2021). One of the most remarkable aspects of ST36 is its ability to activate the vagal-adrenal anti-inflammatory axis. This effect is largely attributed to the stimulation of sensory neurons marked by PROKR2Cre that innervate the deep fascia of the hindlimb (Liu et al., 2021). The significance of this acupoint extends to its profound impact on brain activity, such as the confirmed increase in corresponding electroencephalogram (EEG) power (Li et al., 2011; Yu et al., 2017), or enhanced long-distance connections between hemispheres (Li et al., 2017). Therefore, the choice of ST36 for EEG studies as a biomarker is driven by the desire to uncover specific phenomena related to brain function.

In recent years, virtual reality (VR) has rapidly advanced in medicine, particularly in therapeutic contexts. Therapeutic VR uses immersive tech to manage pain, anxiety, and complex conditions (Chi et al., 2019). A study with 41 chronic neck pain sufferers showed that VR could improve proprioception, lessen cervical joint pain, and decrease related functional limitations (Huang Q. et al., 2022). Research showed that VR-based Cognitive Behavioral Therapy (CBT) worked as well as traditional CBT against the fear of public speaking and outperformed control groups (Gonçalves et al., 2012).

Findings that VR can be a valuable tool in detecting neural patterns have justified the expanded use of VR head-mounted displays in EEG studies (Kim and Biocca, 2018; Choi et al., 2023). This is supported in numerous studies where immersive VR systems were used with EEG while performing imagination of different body movements (Ansado et al., 2021; Hu et al., 2022). Studies also examined the potential of 3D visualization to boost motor-related possibilities in Motor Imagery (MI) (Sollfrank et al., 2015). Results suggest realistic visual feedback in sync with a participant's MI could intensify motor cortex activation, improving Brain-Computer Interface (BCI) intuitiveness for MI-based rehabilitation (Rutkowski, 2016). Immersive VR headsets, known for their deep immersion and personalized virtual scenarios, have the potential to significantly enhance the quality of MI practices that focus on the imagining of specific body movements (Choi et al., 2023).

Traditional acupuncture therapy, often administered in specialized venues under professional guidance, can be time-consuming and resource-intensive for many patients (Lim et al., 2015; Xiong et al., 2022). In the study by Choi et al., volunteers participated in acupuncture sessions under three experimental setups: using their real hand, a synchronized rubber hand, and an asynchronous rubber hand, to investigate the effects on pain tolerance and brain activity. It was found that pain tolerance significantly increased in all conditions. The findings suggest that cognitive components, like visual expectations and body ownership, play a significant role in the analgesic effects of acupuncture (Choi et al., 2022). By integrating VR for a more immersive experience and using EEG to track brain activity, this approach offers a potential breakthrough in acupuncture research and application, especially in the realm of BCI. To our knowledge, this is the first attempt to use VR and EEG in acupuncture. The relative results may provide a new solution for researchers and clinical practitioners using BCI in acupuncture.

As VR technology advances and finds more applications in neuroscience, we've developed a novel VR-based acupuncture method, “Virtual Acupuncture (VA)”. The experiment aimed to investigate whether VA administered at specific time intermissions (e.g., 20 min and 24 h later) could elicit effects comparable to those of Manual Acupuncture (MA) in most participants who experienced MA and subsequently formed neural memories.

## 2 Experimental setup

**Figure 1:**
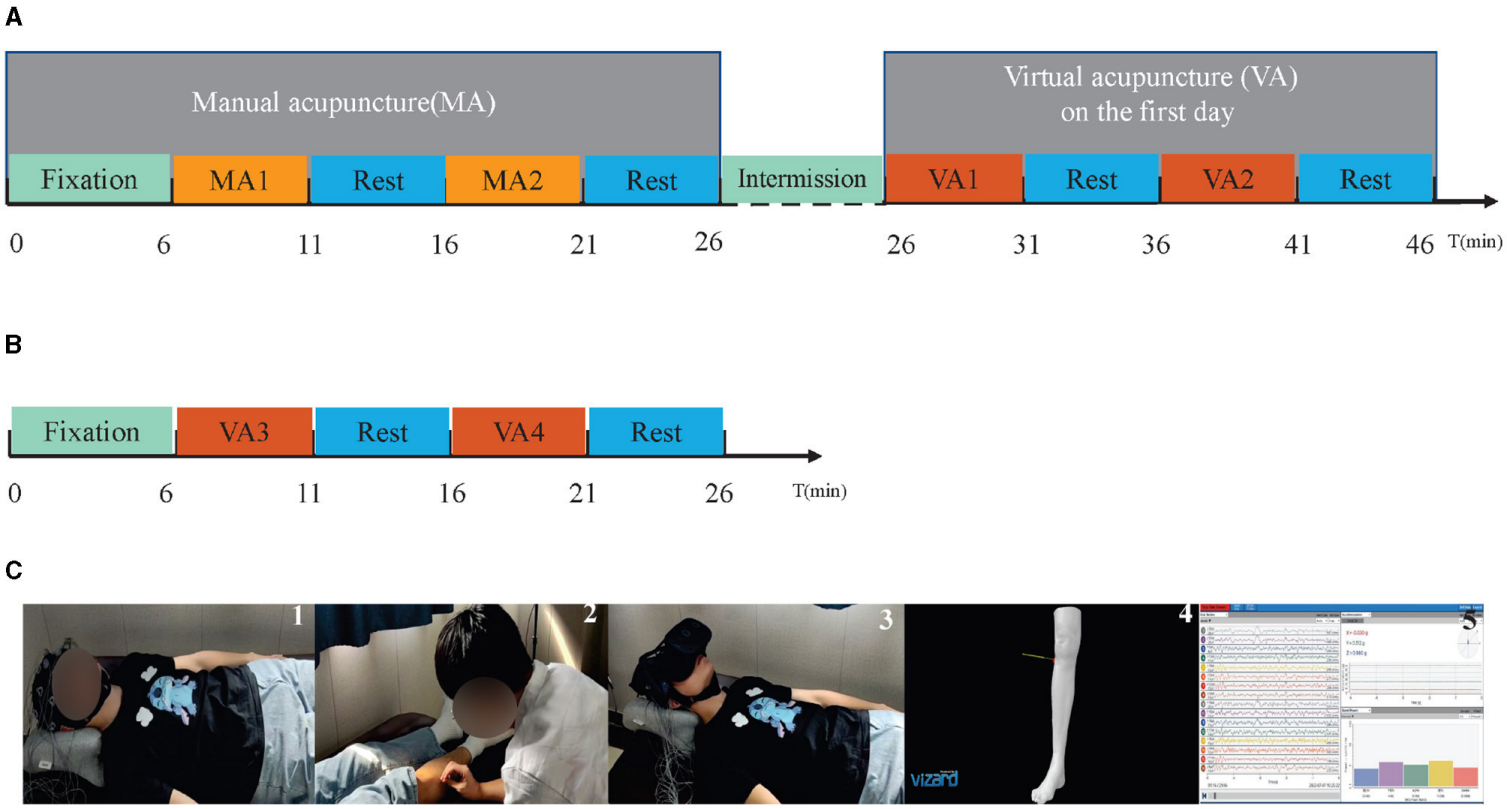
The experiment diagram and several demonstrations. **(A)** Illustrates the experimental procedure for the first day. On this day, the experiment begins at (*t* = 0) with a fixation cue (green bar), which includes 1 min of closed-eye rest and 5 min of open-eye rest. Following this, the MA phase (*t* = 6) involves alternating between two 5-min MA sessions, MA1 and MA2 (orange bar), and two 5-min periods of open-eye rest (blue bar). A 20-min intermission then occurs during which no EEG data is collected. The final stage is the VA phase (*t* = 26), consisting of alternating between two 5-min periods of watching virtual reality acupuncture videos VA1 and VA2 (red bar) and two 5-min open-eye rests (blue bar). **(B)** Depicts the experimental procedure for the second day. On this day, the experiment starts (*t* = 0) with the same fixation cue (green bar) as the first day. Then, the VA phase (*t* = 6) alternates between two 5-min periods of watching virtual reality acupuncture videos VA3 and VA4 (red bar) and two 5-min open-eye rests (blue bar). **(C)** Represents a collection of experimental demonstrations. The first image shows the participant during the fixation cue period. The second image captures the participants during MA. The third image depicts the participant during VA. The fourth image features a sudden scene that appeared during VA. The fifth refers to the display of the signal acquisition interface.

The experiment was conducted at the BCI Laboratory, Nanjing University of Chinese Medicine, Nanjing, China. A total of 24 volunteers were recruited. Two experienced TCM practitioners were responsible for the implementation of manual acupuncture. The Research Ethics Board of the Second Affiliated Hospital of the Nanjing University of Chinese Medicine approved this research, under the IRB number SEZLC20220701. Figure 1 illustrates the experimental process, and the following subsections will provide a detailed explanation for each step.

### 2.1 Experiment preparation

Two actions were implemented before the signal acquisition process: the collection of basic personal information from participants to assess the influence of individual differences on VA effectiveness, and the attainment of signed informed consent forms to validate participants' eligibility. These measures not only ensure ethical compliance but also enable an exhaustive analysis of the study's objectives. The collected information extends beyond age and gender, incorporating primary physiological data such as body temperature, heart rate, and blood pressure (Table 1). To incorporate a more holistic perspective in line with TCM principles, professional TCM practitioners were enlisted to carry out tongue and pulse diagnoses on each participant. These procedures allow insights into the individual's health status from a TCM perspective and enable a better understanding of how these aspects might interact with the effects of VA. Notably, participants received audio cues throughout the experiment in the MA and VA sessions. These prompts mainly functioned as procedural reminders, including, “Acupuncture starts”, “Take a rest”, and “Close your eyes”.

**Table 1:** Subject information table.

**ID**	**Age (year)**	**Sex (M/F)**	**HR^a^ (bpm)**	**BT^b^ (°C)**	**VH^c^ (N/Y)**	**AH^d^ (N/Y)**
Sub01	23	M	82.5	36.8	N	N
Sub02	23	F	93.5	36.2	Y	N
Sub03	23	M	88.5	36.6	Y	Y
Sub04	23	M	80.0	36.6	Y	N
Sub05	23	F	84.0	36.6	N	N
Sub06	22	F	81.0	36.5	Y	N
Sub07	24	M	58.5	36.7	Y	Y
Sub08	25	M	72.5	36.8	Y	N
Sub09	22	M	68.0	36.7	Y	N
Sub10	23	F	86.5	36.6	N	N
Sub11	25	F	86.5	36.6	N	N
Sub12	26	M	62.5	36.5	N	Y
Sub13	19	F	84.5	36.5	Y	Y
Sub14	21	M	66.5	36.5	Y	Y
Sub15	21	M	77.0	36.6	Y	Y
Sub16	19	F	100.5	36.7	N	Y
Sub17	20	F	100.5	37.1	N	N
Sub18	23	M	79.0	36.4	N	N
Sub19	19	F	79.0	36.8	N	N
Sub20	20	F	91.0	36.5	N	N
Sub21	21	F	82.5	36.5	Y	Y
Sub22	23	M	75.5	35.9	Y	N
Sub23	19	F	70.5	36.2	N	N
Sub24	24	M	72.5	36.5	N	N

^a^HR, heartbeats per minute during detection; ^b^BT, forehead temperature during detection; ^c^VH, whether have VR using history; ^d^AH, whether have acupuncture experience.

### 2.2 EEG headset

A 16-channel OpenBCI Gel-Free Electrode Cap Bundle was selected for EEG recording, detailed at https://shop.openbci.com/products/all-in-one-gelfree-electrode-cap-bundle. The EEG headset adheres to the 10-20 international standard for electrode placement and is designed for operation within a normal impedance range of 5–50 *kΩ*, eliminating the need for traditional gel-based application methods. The preparation protocol, involving the soaking of gel-free electrodes in saline solution and their replacement every 20 min, ensures consistent electrode conductivity and the quality of EEG recordings.

**Figure 2:**
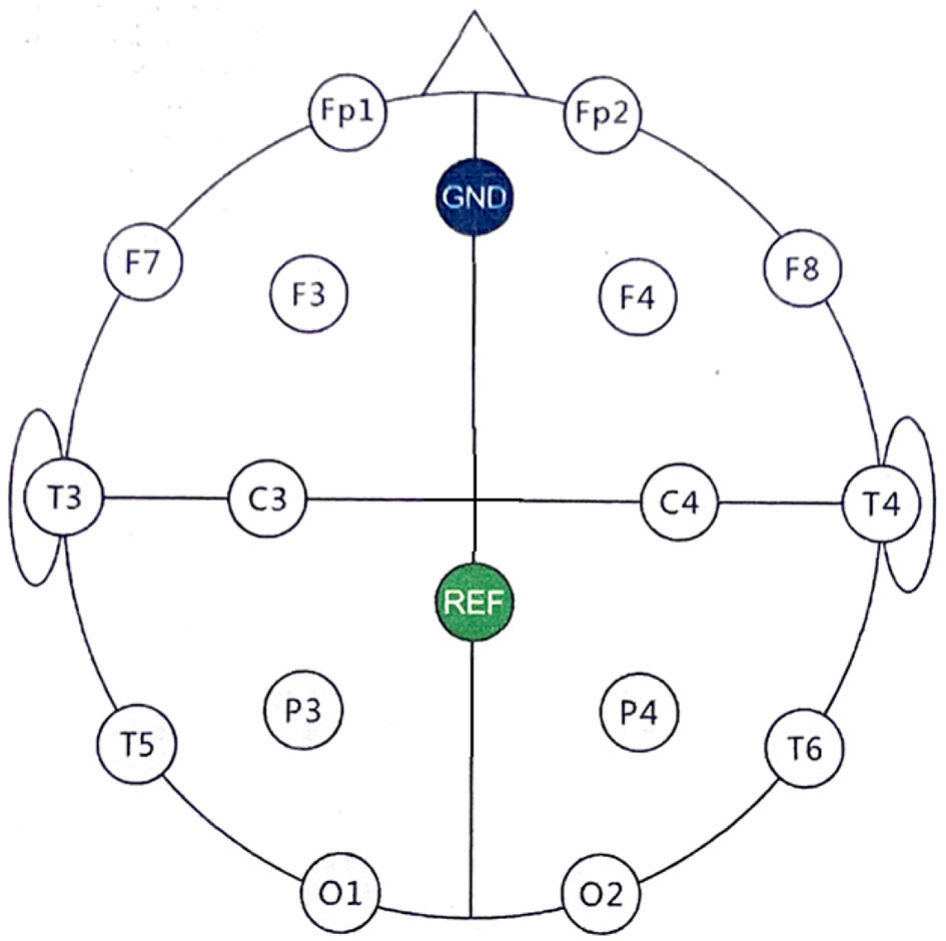
EEG electrode placement using the 10-20 International System. The electrodes are placed as follows: prefrontal cortex (Fp1/Fp2), frontal (F3/F4/F7/F8), temporal (T3/T4/T5/T6), central (C3/C4), parietal (P3/P4), occipital (O1/O2). The GND electrode is positioned on the forehead for grounding, while the REF electrode is centrally placed and acts as the common reference for signal acquisition.

The 16-channel EEG montage is illustrated in Figure 2. The electrodes are evenly distributed covering the entire brain, enabling real-time monitoring of the four brain functional areas, i.e., frontal, parietal, temporal, and occipital lobes. In details, Fp1/Fp2, F3/F4/7/F8, T3/T4/T5/T6, C3/C4, P3/P4, and O1/O2, cover the prefrontal, frontal, temporal, centrally above the motor cortex, parietal, and occipital, respectively. The GND (ground) electrode is placed on the forehead, while the REF (reference) electrode serves as a common reference point for all channels. This configuration is designed to facilitate comprehensive monitoring of brain activity across different areas.

### 2.3 Design of MA

During the MA phase, participants were positioned supine on the acupuncture bed, with the right “Zusanli” exposed, and recorded EEG signals throughout the process. The entire MA process was meticulously planned and divided into five sequential stages with certain cues as mentioned in the previous section. Figure 1A illustrates the experiment diagram. At the beginning of these stages, corresponding audio cues were provided. These stages were designed to establish foundational measures, execute the treatment, and allow periods of rest in between. The intention was to create a structured environment to discern the effects of the acupuncture intervention.

The first stage involved a period of fixation hint, composed of 1-minute closed-eye rest and 5-minute open-eye rest. This period served as an opportunity to record EEG readings that were not affected by acupuncture, which are critical for later comparison and understanding of the brain's electrical activity changes due to the intervention. Throughout this six-minute phase, participants were guided to relax as the equipment captured the essential EEG data.

After the fixation hint, participants alternated between receiving acupuncture and having open-eye rests twice, each lasting 5 min. During the open-eye rest stages, participants were instructed to keep their eyes open while remaining at rest. These intermissions provided brief recovery periods between treatments and tracked any physiological changes in the participants due to either the acupuncture treatment or the simple passage of time. A professional acupuncturist performed acupuncture on the “Zusanli” acupoint.

This structure aims to control as many variables as possible, ensuring that the changes recorded are due to the acupuncture treatment and not confounding factors. This format also facilitates monitoring participants' responses over a standardized timeline.

### 2.4 Design of VA

In the VA events, participants also lie supine on an acupuncture bed, undergoing the standard procedure. The VA events are intended to replicate the MA experience in a virtual environment, using the HTC VIVE Pro 2's head-mounted display, two BASE STATION 2.0 units, and the necessary cables.

The VA video, developed on the Vizard platform, is designed to mimic a realistic acupuncture scenario. It incorporates various visual elements and necessary auditory elements to meet the VA requirements. The visual components include a pitch-black background for enhanced concentration, a model of one leg, and an acupuncture needle. A prompt for the “Zusanli” acupoint appears for 3 s, showing the needle penetrating the acupoint and the color of the acupoint transitioning from red to blue. During rest periods, the background reverts to pitch black. The auditory components of the video include key broadcast information during each acupuncture phase to simulate the participants' sensations of MA.

To examine the potential effects of MA on VA, the VA events were conducted over 2 days. On the first day, after a 20-minute break post-MA, two stages of VA (VA1 and VA2) were administered. Each stage involved 5 min of virtual stimulation and a 5 min rest period. This sequence was repeated once, as depicted in Figure 1A. This duration was chosen twenty-minute intermission for practical needs, i.e., experimental setup, EEG electrode connection, etc. In detail, during this intermission, the researchers replenished the saline in the gel electrodes of the EEG acquisition device to ensure high-quality data collection. Additionally, this time was used to set up the virtual reality equipment for the subsequent VA session and to allow participants adequate time to alleviate any sensations from MA.

The VA sessions on the second day were carefully designed to closely mirror the MA sessions, as depicted in Figure 1B and structured into five sequential stages. A fixation hint was presented to guide participants' attention, and this stage lasted for 6 min. Subsequently, VA3 was administered for 5 min, followed by a 5-minute rest period. After the rest period, VA4 was conducted for another 5 min, followed by an additional 5 min of rest. Each stage served a specific purpose in the VA procedure, ensuring a systematic and well-defined protocol for the participants.

The inclusion of VA offers an optional alternative treatment to understand whether it is more effective in conjunction with MA or when used separately. One advantage of this design is that it allows for objective and trackable assessment of the stimulation effects on participants.

## 3 Methodology

**Figure 3:**
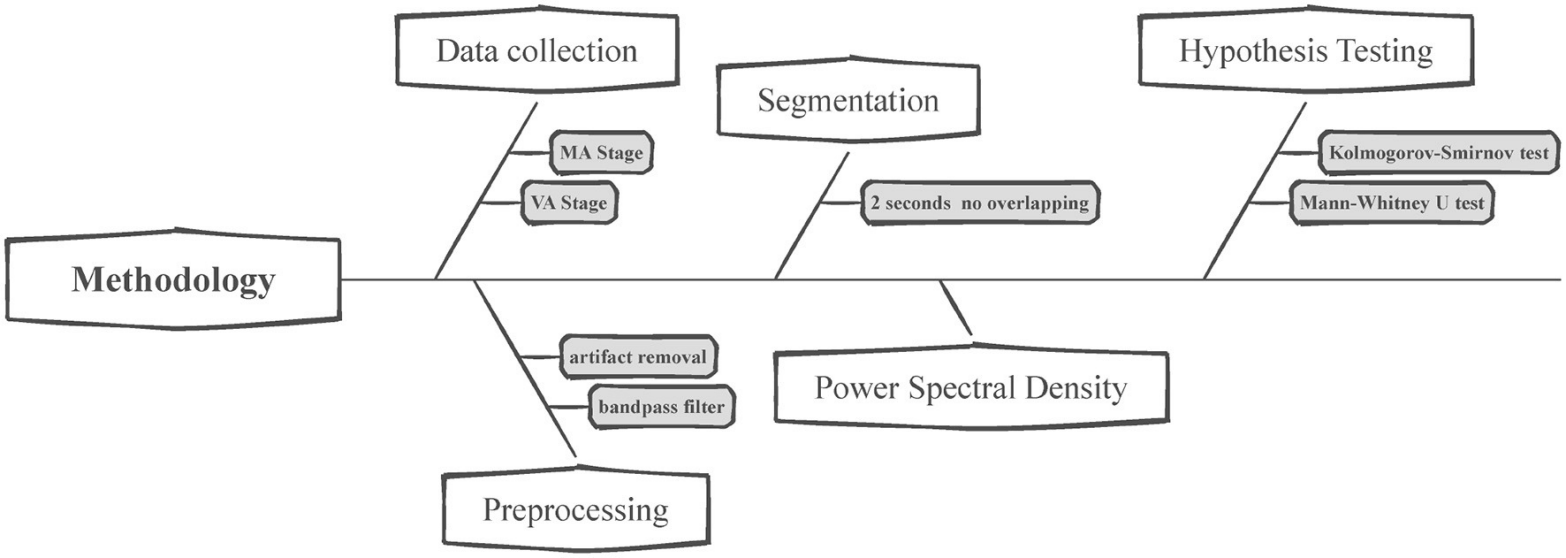
Flowchart for EEG data analysis. This figure presents the structured methodology for analyzing EEG data collected during the manual and virtual acupuncture treatment. The process begins with data collection, where EEG is recorded during both the MA and VA stages. Preprocessing follows, which includes artifact removal and bandpass filtering to clean the data. After segmenting the preprocessed data into 2-s non-overlapping intervals, the Power Spectral Density (PSD) of these segments is calculated. Lastly, Hypothesis testing is conducted using statistical methods such as the Kolmogorov-Smirnov test and the Mann-Whitney *U*-test to evaluate the EEG data and determine the significant effects of VA.

The EEGs of 24 subjects were recorded during the MA and VA stages, and Figure 3 depicts the procedure for data collection and preprocessing, segmentation, feature extraction, and hypothesis testing. The EEG signals were initially preprocessed to remove any artifacts, after which they were segmented into smaller segments. Subsequently, the channel-weighted frequency power summation of each EEG segment was calculated to prepare for the Mann-Whitney *U*-test. The following sections will explain further details regarding each module and its functionalities.

### 3.1 Data collection

Two experienced EEG readers were responsible for EEG data collection in this experiment. During the data collection phase, EEG signals from 24 subjects were recorded across six different events i.e., MA1, MA2, VA1, VA2, VA3, and VA4, over 2 days. Each participant's EEG data were collected for a total duration of 72 min, equating to 4,320 s. The electrodes used were strategically placed to cover the entire brain, ensuring comprehensive real-time monitoring of the four principal brain functional areas: the frontal, parietal, temporal, and occipital lobes. This setup facilitated a thorough investigation of the neural activities associated with both manual and virtual acupuncture treatments.

### 3.2 Preprocessing

The EEG data underwent bandpass filtering within the [0.5, 50]*Hz* frequency range, applying a Finite Impulse Response (FIR) filter with a Hamming window, following EEGLAB's standards. This high-order filter (order of 4142) was chosen to effectively suppress high-frequency artifacts and adjust for DC offset, ensuring the data's fidelity for further analysis. The use of FIR filters aligns with EEGLAB's approach to avoid phase distortion and maintain signal integrity.

**Figure 4:**
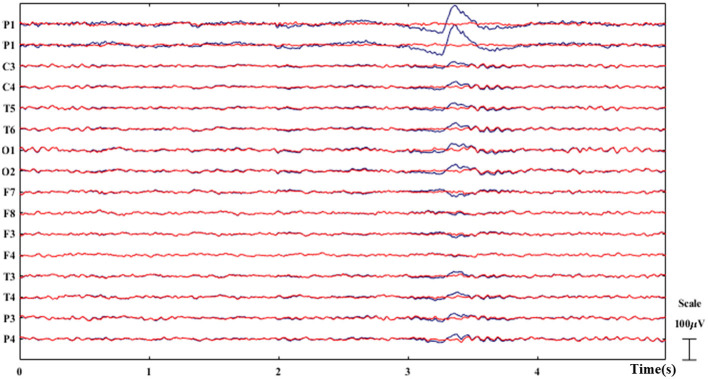
Example of ICA-based artifact removal. The original EEG is plotted in blue while the clean EEG after artifact removal is plotted in red. The eye blinks are successfully removed as demonstrated.

In our experiment, two experienced EEG readers implemented the ICA to ensure the ICs removal would not impact the primary findings. The raw EEG data from each subject was decomposed into 16 independent components and the number of ICs removal was restricted to <2. As illustrated in Figure 4, ICA effectively removed the eye blinks while preserving the clean EEG. This selective removal of contaminated ICs allows for the preservation of the integrity of the underlying neural signals, thereby enhancing the reliability of our EEG data analysis and the validity of subsequent interpretations. The ICA component rejection followed the ICA artifact removal instruction of EEGLAB2022.1 (https://eeglab.org/tutorials/06_RejectArtifacts/RunICA.html).

### 3.3 Segmentation

**Figure 5:**
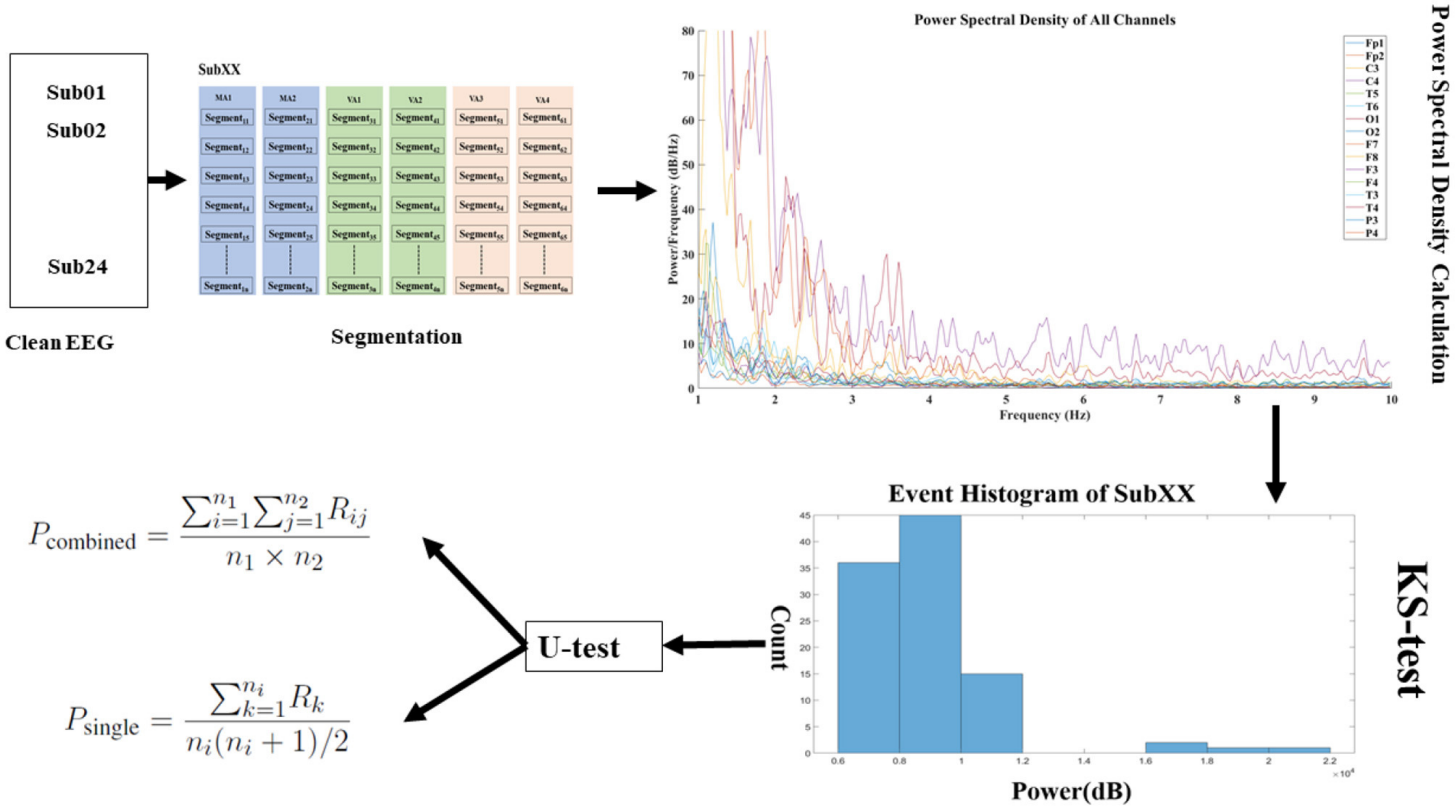
Data segmentation and analysis flowchart. The clean EEG data is segmented into events using sliding window techniques, and the weighted frequency power for each channel within these windows is calculated. In the power spectral density graph, the *x*-axis signifies frequency, and the *y*-axis indicates power per frequency with various colored lines representing different channels. Prior to applying for the *U*-test, the *KS*-test is conducted to verify the normal distribution of the data, ensuring the appropriateness of subsequent statistical analyses. Subsequently, *U*-test is performed to assess the statistical significance between the conditions labeled as VA and MA, providing insight into the differences in brain activity under these two states.

Figure 5 depicts the process of EEG data segmentation, Power Spectral Density (PSD) calculation, and statistical testing for analysis. Over 2 days, participants collected EEG data from six events: MA1, MA2, VA1, VA2, VA3, and VA4 (Figures 1A, B). Each event lasted 5 min and is denoted as *v* (*v* ∈ [1, 6]). The dataset can be represented as $D=\left\{X_{1}^{1},X_{1}^{2},\ldots ,X_{j}^{v}\ldots ,X_{24}^{5},X_{24}^{6}\right\}$, where *j* ∈ [1, 24] represents the ID of participant.

To analyze the data, each event's data is segmented using a sliding window of 2 s with no overlapping. This results in segments denoted as $X_{j}^{v}=\left\{\psi_{1},\psi_{2},\ldots ,\psi_{n}\right\}$, where *n* ∈ [1, *N*]. Here, *N* represents the total number of segments the data is divided into.

This segmentation approach allows for a more detailed EEG data analysis, enabling the examination of specific time intermissions within each event. By dividing the data into segments, we can explore different patterns and features in the EEG signals, facilitating a better understanding of the similarities and differences between MA and VA events.

### 3.4 Power spectral density

Channel-weighted frequency power summation is a technique that emphasizes the significance of various frequency bands by multiplying the PSD by its frequency and aggregating the results across channels, capturing both their importance and spatial distribution. The PSD for every channel in segment *t*_*n*_ is calculated as:


(1) $$\begin{array}{c} p_{n,f}^{m}=|\frac{1}{Q}\overset{Q-1}{\underset{k=0}{\sum }}w\left(k\right)\psi_{n}^{m}\left(k\right)e^{-j2\pi fkT}{|}^{2} \end{array}$$

where: $\psi_{n}^{m}\left(k\right)$ represents the *k*-th sample of the *m*-th channel in segment ψ_*n*_, *Q* is the length of the segment, *w*(*k*) is the windowing function applied to the segment, *f* ∈ [1, 50] is the frequency variable, *T* is the sampling period. $p_{n,f}^{m}$ is the power at frequency *f* for the segment *t*_*n*_ of the *m*-th channel.


(2) $$\begin{array}{c} S_{n}=\frac{1}{M}\overset{M}{\underset{m=1}{\sum }}\underset{f\in F}{\sum }f\times p_{n,f}^{m} \end{array}$$

where: *M* is the total number of channels, *F* is the set of all frequencies for computed the PSD, *S*_*n*_ is a measure designed to quantify the weighted spectral content with a segment.

Building on this, the dataset can represented as $X_{j}^{v}=\left\{S_{1},S_{2},\ldots ,S_{n}\right\}$, where *n* ∈ [1, *N*], provides a comprehensive representation of both the frequency and spatial dimensions. It particularly emphasizes frequency bands that are often associated with specific physiological or cognitive states.

### 3.5 Hypothesis testing

For statistical analysis, especially with large datasets obtained from the sliding window method, the Mann-Whitney *U*-test (*U*-test) is used. The choice of this non-parametric test was informed by the Kolmogorov-Smirnov test (*KS*-test), which indicated that only a few groups conformed to the normal distribution. By leveraging the *U*-test, we achieved robust statistical validation, ensuring a meaningful analysis of the extensive dataset (Nachar, 2008).

#### 3.5.1 Kolmogorov-Smirnov test

The fundamental principle of the *KS*-test is to compare the difference between a sample's empirical cumulative distribution function (ECDF) and the theoretical distribution's cumulative distribution function (CDF), where the theoretical distribution in this context is the normal distribution. The *KS*-test statistic *D* is defined as the maximum absolute difference between the sample's ECDF and the theoretical CDF as in Equation (3):


(3) $$\begin{array}{c} D=max_{x}|F_{n}\left(x\right)-F\left(x\right)| \end{array}$$

Here, *F*_*n*_(*x*) is the sample's ECDF, *F*(*x*) is the theoretical cumulative distribution function (in the case of a normality test, this is the CDF of the normal distribution), and *x* represents the observed values, which in this paper refers to the result *S*_*n*_ from Equation (2).

The results of the KS-test are shown in Table 2, where bold black text indicates a *p*_*value* ≥ 0.05, meaning that the PSD for that event conforms to a normal distribution. The character “-” indicates *p*_*value* <0.05, meaning that the event does not satisfy normal distribution. Table 2 reveals that only 10 out of 192 events satisfy a normal distribution, hence, in the subsequent tests, we chose to use the *U*-test, which does not require the assumption of a normal distribution.

**Table 2:** P_value with *KS*-test for the 24 subjects.

**ID**	**MA1**	**MA2**	**VA1**	**VA2**	**VA3**	**VA4**
Sub01	–	–	–	–	–	–
Sub02	–	**0.07**	–	–	–	–
Sub03	–	–	–	–	–	–
Sub04	–	–	–	–	–	–
Sub05	–	–	–	–	–	–
Sub06	–	–	–	–	–	–
Sub07	–	–	–	–	–	–
Sub08	–	–	–	–	–	–
Sub09	**0.19**	–	–	–	–	–
Sub10	–	**0.14**	–	**0.58**	–	–
Sub11	–	–	–	–	–	–
Sub12	–	–	–	–	**0.49**	–
Sub13	–	–	–	–	–	–
Sub14	–	–	–	–	–	–
Sub15	–	–	–	–	**0.33**	**0.28**
Sub16	–	–	–	–	–	–
Sub17	–	–	–	–	–	**0.10**
Sub18	–	–	–	–	–	–
Sub19	–	–	–	–	–	–
Sub20	–	–	–	–	–	–
Sub22	–	–	–	–	–	**0.09**
Sub23	–	–	–	–	**0.24**	–
Sub24	–	–	–	–	–	–

The bold text indicates a normal distribution of events.

#### 3.5.2 Mann-Whitney *U*-test

The first type of analysis involved comparing the combined sets of MA events MA1, MA2 with the combined sets of VA events VA1, VA2, VA3, VA4. This *U*-test can be represented as shown in Equation (4). Where *P*_combined_ represents the *p* − *value* for the *U*-test comparing the combined MA events with the combined VA events. *R*_*ij*_ denotes the rank assigned to the *i*-th MA event compared to the *j*-th VA event. *n*_1_ and *n*_2_ indicate the number of observations in the MA and VA groups, respectively.


(4) $$\begin{array}{c} P_{\text{combined}}=\frac{\sum_{i=1}^{n_{1}}\sum_{j=1}^{n_{2}}R_{ij}}{n_{1}\times n_{2}} \end{array}$$

The second type of analysis involved conducting individual U-tests between each MA event and its corresponding VA event. For the *i*-th pair of MA and VA events, the U-test was performed, and the resulting *p* − *value* can be calculated using Equation (5). Where, *R*_*k*_ represents the rank assigned to the *k*-th observation in the paired MA and VA events, and *n*_*i*_ denotes the number of observations in that specific pair.


(5) $$\begin{array}{c} P_{\text{single}}=\frac{\sum_{k=1}^{n_{i}}R_{k}}{n_{i}\left(n_{i}+1\right)/2} \end{array}$$

*P*_combined_ and *P*_single_ provide a statistical measure of the significance of the differences between the MA and VA events, both when combined and individually for each pair. The data processing, channel-weighted frequency power estimation, and U-test were performed using the MATLAB R2021b platform.

## 4 Results

The significance of ST36, an acupuncture point, is particularly noteworthy in the context of its influence on neurological functions (Huang H. et al., 2022). This acupoint's profound impact on brain activity can be observed and tracked using EEG. Consequently, selecting ST36 for EEG studies as a biomarker is motivated by the objective of exploring specific phenomena related to brain function. To further understand this, the results of the *U*-test, as detailed in Table 3, compare the EEG signals between VA and MA. Table 4 illustrates different frequency bands' impact on VA and MA. The following analysis provides insights into the similarities and differences between acupuncture methods and relative EEG activities.

**Table 3:** The *U*-test results (p-value) of the 24 subjects.

**ID**	**Sex (M/F)**	**MA_T^a^ vs. VA_T^b^**	**MA1 vs. VA1**	**MA1 vs. VA2**	**MA1 vs. VA3**	**MA1 vs. VA4**	**MA2 vs. VA1**	**MA2 vs. VA2**	**MA2 vs. VA3**	**MA2 vs. VA4**
Sub01	M	0.02	0.02	**0.56**	**0.22**	**0.12**	0.04	**0.05**	**0.77**	**0.04**
Sub02	F	**0.30**	**0.08**	0.01	**0.72**	**0.33**	**0.35**	**0.41**	**0.12**	**0.07**
Sub03	M	**0.15**	0.01	**0.56**	0.03	0.04	**0.28**	**0.19**	0.03	**0.60**
Sub04	M	**0.97**	**0.38**	**0.31**	**0.31**	**0.81**	**0.21**	**0.60**	**0.73**	**0.56**
Sub05	F	**0.64**	**0.27**	**0.65**	**0.69**	**0.24**	**0.27**	**0.80**	**0.31**	**0.83**
Sub06	F	**0.07**	0.00	0.04	**0.09**	**0.22**	0.00	0.04	**0.09**	**0.34**
Sub07	M	0.02	0.03	**0.05**	**0.05**	**0.07**	0.03	0.00	0.01	**0.06**
Sub08	M	0.00	0.00	0.00	0.00	0.00	0.00	0.00	0.00	0.00
Sub09	M	**0.17**	**0.42**	**0.37**	**0.48**	**0.40**	**0.45**	**0.12**	**0.08**	**0.45**
Sub10	F	**0.20**	**0.31**	**0.23**	**0.47**	**0.33**	**0.29**	**0.30**	**0.03**	**0.33**
Sub11	F	**0.06**	**0.10**	**0.16**	0.04	0.00	**0.28**	**0.44**	0.00	**0.08**
Sub12	M	**0.06**	0.04	**0.07**	**0.06**	**0.12**	0.04	0.03	0.03	**0.88**
Sub13	F	**0.26**	**0.39**	**0.34**	**0.45**	**0.08**	**0.36**	**0.33**	**0.46**	**0.08**
Sub14	M	**0.17**	**0.06**	0.03	**0.64**	**0.13**	**0.06**	0.03	**0.42**	**0.18**
Sub15	M	**0.16**	0.03	**0.57**	0.00	0.03	**0.17**	**0.33**	0.04	**0.28**
Sub16	F	**0.10**	**0.10**	**0.15**	**0.12**	**0.13**	**0.10**	**0.16**	**0.13**	**0.16**
Sub17	F	0.00	0.00	0.00	0.00	0.00	0.00	0.00	0.00	0.00
Sub18	M	**0.57**	**0.19**	**0.32**	**0.35**	**0.44**	**0.76**	**0.94**	**0.94**	**0.83**
Sub19	F	**0.33**	**0.69**	**0.65**	**0.68**	**0.68**	**0.14**	**0.13**	**0.11**	**0.11**
Sub20	F	**0.70**	0.04	**0.83**	**0.08**	**0.08**	0.03	0.04	**0.79**	**0.50**
Sub21	F	0.03	0.03	0.04	0.00	0.03	**0.25**	**0.11**	**0.15**	**0.05**
Sub22	M	0.02	0.03	0.03	**0.09**	0.03	**0.05**	0.03	**0.09**	**0.06**
Sub23	F	0.01	0.00	0.01	0.02	0.00	0.02	0.01	0.02	0.00
Sub24	M	0.04	0.04	0.04	0.03	0.03	**0.66**	**0.05**	0.00	**0.09**

^a^MA_T: MA1 and MA2.

^b^VA_T: VA1, VA2, VA3 and VA4.

The bold text highlights that there is no significant difference between MA and VA.

**Table 4:** P-value for the comparison between MA_T^a^ and VA_T^b^ across different bands for the 24 subjects.

**ID**	**Raw > 1 Hz**	**Delta 1–4 Hz**	**Theta 4–8 Hz**	**Alpha 8–13 Hz**	**Beta 13–30 Hz**	**Gamma > 30 Hz**
Sub01	0.02	0.00	0.00	0.00	0.00	0.00
Sub02	**0.30**	**0.27**	**0.06**	0.00	0.00	0.00
Sub03	**0.15**	0.01	0.00	0.03	0.00	**0.77**
Sub04	**0.97**	**0.24**	**0.28**	**0.76**	**0.87**	**0.61**
Sub05	**0.64**	**0.46**	**0.41**	**0.33**	0.00	0.00
Sub06	**0.07**	**0.49**	**0.52**	**0.48**	**0.76**	**0.50**
Sub07	**0.20**	**0.49**	**0.42**	**0.42**	**0.58**	**0.65**
Sub08	0.00	0.00	0.00	0.00	0.00	0.00
Sub09	**0.17**	**0.42**	**0.64**	**0.15**	0.04	**0.44**
Sub10	**0.20**	**0.55**	**0.36**	0.01	**0.36**	**0.06**
Sub11	**0.06**	0.04	0.02	0.03	0.01	0.01
Sub12	**0.06**	**0.35**	**0.16**	**0.21**	0.00	0.00
Sub13	**0.26**	**0.15**	**0.89**	**0.30**	**0.30**	**0.82**
Sub14	**0.13**	**0.47**	**0.25**	0.02	0.02	0.03
Sub15	**0.05**	**0.11**	0.02	0.01	0.01	0.01
Sub16	**0.37**	**0.09**	**0.10**	**0.13**	**0.13**	0.01
Sub17	0.00	0.00	0.00	0.00	0.00	0.00
Sub18	**0.63**	**0.52**	**0.60**	**0.48**	**0.48**	**0.18**
Sub19	**0.99**	**0.09**	**0.29**	**0.45**	**0.32**	**0.31**
Sub20	**0.08**	0.02	0.01	0.01	0.01	0.02
Sub21	0.03	**0.06**	0.01	0.01	0.01	0.00
Sub22	0.02	0.00	0.01	0.00	0.00	0.00
Sub23	0.01	**0.55**	**0.58**	**0.71**	**0.71**	0.00
Sub24	**0.72**	**0.92**	**0.31**	**0.33**	**0.33**	**0.07**
**Similarity** ^c^		**79.17%**	**79.17%**	**70.83%**	**54.17%**	**62.50%**

^a^MA_T: MA1 and MA2. ^b^VA_T: VA1, VA2, VA3 and VA4. ^c^Similarity: see Equation (7) for details.

The bold text highlights that there is no significant difference between MA and VA.

The comparison between the effects of VA and MA on EEG signals has provided insightful data as shown in Table 3, suggesting that VA holds promise as a non-invasive alternative to traditional acupuncture methods. The values with bold text indicate *p* − *value* ≥ 0.05, signifying that there is no statistically significant difference between VA and MA conditions. Conversely, values in normal text represent *p* − *value* <0.05, which statistically indicates a significant difference between the VA and MA conditions. The analysis began by consolidating the stages of MA and VA into two groups: MA_T (encompassing MA1 and MA2) and VA_T (including VA1, VA2, VA3, and VA4). The results showed that for 16/24 = 66.67% of the participants, as listed in the 3rd column of Table 3. There was no significant difference in EEG signals between MA_T and VA_T, suggesting that VA could induce brain activities comparable to MA.

Further examination of the individual stages of MA and VA revealed that in 122/192 = 63.54% of comparisons, EEG signals from VA stages did not significantly differ from those of MA stages, indicating considerable consistency across subjects. Specifically, for 7/24 = 29.17% of the subjects, none of their VA stages significantly differed from MA stages, and for 21/24 = 87.50% of the subjects, more than 50% of VA and MA events were consistent. This suggests that VA could potentially serve as an effective alternative to MA, particularly for individuals who might be apprehensive or physically unable to undergo MA.

To investigate the specific impact locations of acupuncture, each segment, $\psi_{n}^{m}\left(k\right)$ obtained from Equation (1), is multiplied by its corresponding frequency value for each *f*, within the PSD $p_{n,f}^{m}$, then average this across all segments within an event, as shown in the following equation:


(6) $$\begin{array}{c} S_{m}=\frac{1}{N}\overset{N}{\underset{n=1}{\sum }}\underset{f\in F}{\sum }f\times p_{n,f}^{m} \end{array}$$

where: *N* is the total number of segments in *v*-th event, *F* ∈ [1, 50] is the set of all frequencies for computed the PSD, *S*_*m*_ represents the weighted spectral content of the *m*-th channel within the *v*-th event.

The Supplementary material primarily displays the topography for 24 subjects, with the values in the graphs representing *S*_*m*_ under the condition that *f* ∈ [1, 50]*Hz*. The specific calculation method for *S*_*m*_ is outlined in Equations (1, 6).

**Figure 6:**
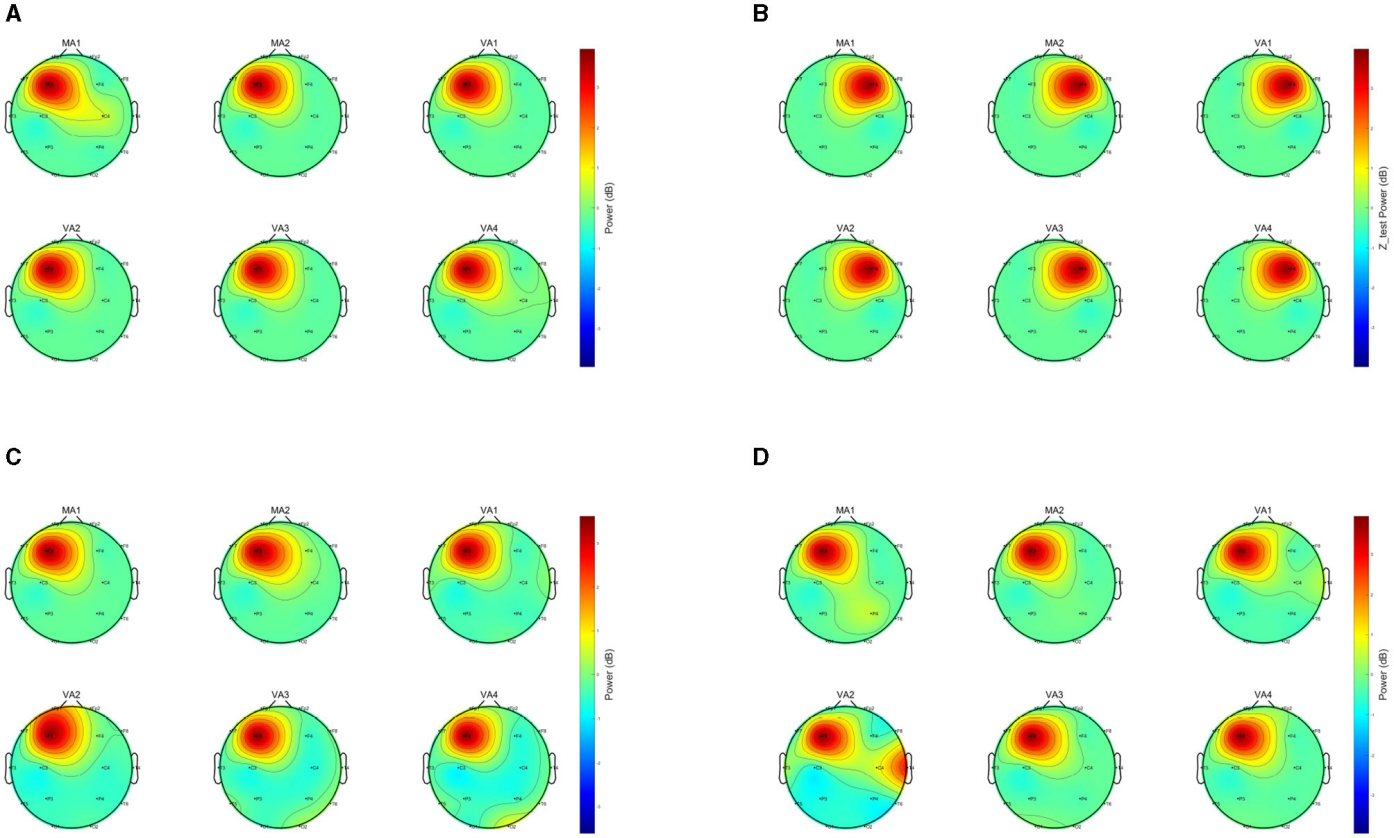
Comparison of EEGs under MA and VA. The image presents a collection of EEG topographies. Each topography corresponds to a distinct event and illustrates the weighted spectral content *S*_*m*_, as computed by Equation (6). The subplots are labeled MA1, MA2, VA1, VA2, VA3, VA4, showing power distribution across the scalp. Power levels are denoted by the color bar scale in decibels (dB). Vibrant central colors mark areas of high power, and the perimeter's cooler colors denote areas of low power. **(A)** Topography of Sub04. **(B)** Topography of Sub09. **(C)** Topography of Sub18. **(D)** Topography of Sub19.

In our investigation of the effects at the Zusanli point, we registered notable brain activity during both the MA and the VA across various individuals. Remarkably, in 10 out of the subjects studied, the brain responses in the VA stages showed no significant difference from those in the MA stages. Within this group, four individuals displayed brain activations that were localized specifically to the frontal lobe regions throughout their sessions. In Figure 6 subjects Sub04, Sub09, Sub18, and Sub19 displayed activation within the frontal lobe region. These frontal regions are frequently associated with the cognitive processing of pain, and sensory input, and could also relate to the anticipatory effects of acupuncture therapy (Zhu et al., 2021). This congruence in activation sites further supports the capability of VA to mirror the neural activations induced by MA, highlighting its potential as a viable, non-invasive alternative in acupuncture practice.

Despite the significant overlaps in EEG signal patterns between VA and MA, the study also acknowledges instances of divergence, observed in 59/192 = 36.46% of comparisons, suggesting that VA may not entirely simulate the impact of MA for some individuals. This variability underscores the importance of personalizing acupuncture treatments, taking into account individual physiological and psychological differences that might influence the brain's response to VA.

## 5 Discussion

To our best knowledge, this is the very first attempt to use VR for acupuncture stimulation, coupled with EEG as an indicator. With the proposed method, acupuncture can be implemented objectively, and the neural stimulation effects can be tracked objectively, to overcome the expert bias in traditional manual acupuncture. The subsequent subsections will further discuss the results of VA from the perspectives of gender, duration, and frequency band. Additionally, the limitations of this study and future work will be addressed.

### 5.1 VA efficiency variation across days

Table 3 reveals notable temporal variations in the effects of VA, with significant differences emerging at various time points following the completion of MA. Specifically, when VA was performed 20 min after the completion of MA, 57/96 = 59.36% of the EEG signals from VA did not show significant differences compared to MA. However, when VA was performed 24 h after the completion of MA, the proportion of non-significant differences increased to 65/96 = 67.71%.

**Figure 7:**
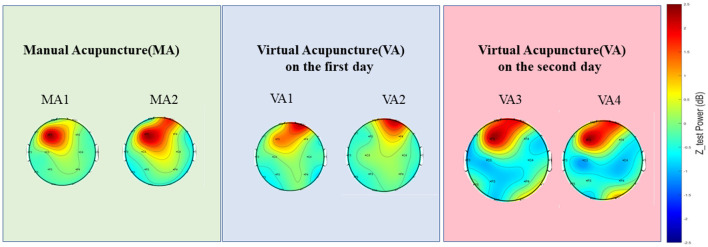
A demonstration (Sub06) of the EEGs of different events across days. Topographies showing the power spectral density distribution across the scalp during MA sessions (MA1 and MA2) and VA sessions on the first (VA1 and VA2) and second day (VA3 and VA4). The color scale represents the power in decibels (dB), with warmer colors indicating higher power and cooler colors indicating lower power. These topographies allow for the visualization of the brain's electrical activity and its modulation by acupuncture in various sessions.

For instance, in the case of Sub06 (Figure 7), during the MA stage, there was activation in the right frontal region and some weak inhibition in the central areas. However, in this subject's VA1 and VA2 stages, only activation in the right frontal region was observed without inhibition in the main areas. On the other hand, both VA3 and VA4 stages exhibited noticeable effects.

The efficacy of VA may vary depending on the intermission after the MA session. As time progresses post-MA, the potential for VA to mimic the effects of MA might increase. This variation in effectiveness could be attributed to several factors. Firstly, the peak effects of MA on brain activity might not be immediate but could occur after a certain duration (Li et al., 2014). Secondly, the brain may need time to adapt and respond fully to the stimuli provided by MA (Yan et al., 2022).

### 5.2 Gender differences in VA efficiency

Table 3 shows significant differences between males and females in the EEG signals from VA to MA. Specifically, 58/96 = 60.41% of the male data didn't show significant differences between VA and MA, the corresponding proportion for females was 64/96 = 66.67%. However, this slight increase in proportion does not suggest that gender is a significant factor affecting the outcomes of VA and MA.

In summary, the minor discrepancies in the effects of VA across genders are less reflective of broad gender-based differences and more indicative of individual variability. This observation aligns with the concept that VA may be a universally applicable alternative to MA, effective across gender lines. Despite this, some studies have shown that physiological and psychological factors, which can vary significantly between males and females, may influence the brain's response to pain and treatments like acupuncture (Qiu et al., 2010). Hormonal differences and the neural networks involved in emotion and cognition could contribute to these observed variations (Lund and Lundeberg, 2008). However, the overarching data suggest that VA has the potential to be a gender-neutral treatment, with the effectiveness of acupuncture not predominantly dictated by gender but rather by the individual's unique physiological and psychological makeup.

### 5.3 VA efficiency variation across bands

In the analysis of EEG data across various frequency bands in Table 4, text formatting is essential for interpretation. Bold text for *p* − *value* over 0.05 indicates no significant differences between VA and MA conditions in the specific frequency band. Regular text for *p* − *value* under 0.05 suggests significant differences between the two. The “Raw” in the second column of Table 4 refers to EEG data that have been processed through filtering, ICA, and segmentation, yet have not been analyzed across specific frequency bands. This data covers the full frequency range of [1, 50]*Hz*.

The analysis revealed that the Delta (1–4 *Hz*) and Theta (4–8 *Hz*) bands had notable counts of non-significant differences between VA and MA outcomes, with 17 out of 24 subjects for Delta and 15 out of 24 for Theta showing no significant difference. These findings suggest that the Delta and Theta bands are more representative of the overall brain activity patterns in the sample, a conclusion that is in line with the research conducted by Yu et al. Their study highlighted significant changes in clustering coefficients and path lengths in these bands during and post-acupuncture (Yu et al., 2021). Furthermore, the study's detailed examination of brain oscillations, particularly focusing on the Delta and Theta bands, provides valuable insights into the brain's response to acupuncture, as these frequencies are crucial in the brain's electrical activity and are often linked to states of relaxation and meditation (Herrmann et al., 2016).


(7) $$\begin{array}{c} similarity=\frac{AG+AL}{AN} \end{array}$$

Where *AG* represents the number of subjects for whom a specific frequency band and the raw data both exhibited significant differences between MA_T and VA_T. *AL* denotes the number of subjects for whom a specific frequency band and the raw data both did not exhibit significant differences between MA_T and VA_T, indicating consistency in brain activity patterns across these acupuncture techniques. *AN* is the total number of participants involved in the experiment, which currently stands at 24.

Reflecting on the relationship between segmented frequencies and raw data, it becomes clear that the Delta and Theta bands significantly reflect the brain's overall activity in response to acupuncture. Analysis from Table 4 reveals that in the Theta and Delta band, 19 out of 24 subjects showed either significant or non-significant differences that aligned with those observed in the raw data, representing a 79.17% consistency rate.

These findings highlight a substantial portion of subjects for whom VA and MA treatments generate comparable brain activity patterns within these pivotal frequency bands. This observation underlines the capacity of the Delta and Theta bands to accurately represent the broader brain responses to acupuncture interventions. Such insights, resonating with prior studies, emphasize the critical role of the Delta and Theta bands in the brain's electrical activities, especially those associated with states of relaxation and meditation. This concurrence with the raw data not only underscores the importance of these bands in reflecting the brain's acupuncture response but also strengthens the study's conclusions, aligning them with established scientific literature.

### 5.4 Limitations of this work

A low-density EEG headset is one limitation of this work. There are 3 reasons for using a 16-channel OpenBCI EEG headset in this study. Firstly, the electrodes are evenly distributed covering the entire brain, enabling real-time monitoring of the four brain functional areas, i.e., frontal, parietal, temporal, and occipital lobes, in the experiment. Secondly, aside from the preparation, questioning, cleaning, and other procedures, the experiment takes nearly an hour. Since acupuncture at ST36 requires participants to adopt a supine position, the high-channel electrodes significantly affect the wearing comfort, potentially impacting acupuncture's stimulation effect. In contrast, low-channel headsets have a clear advantage in terms of comfort during wearing. Lastly, low-channel headsets facilitate the midway replenishment of saline solution to poorly contacted electrodes during the experiment, avoiding data loss.

However, the 16-channel EEG headset can only guarantee a limited spatial resolution, thereby potentially limiting our ability to deeply understand the specific brain mechanisms activated by VA. This provides us with a certain hint for subsequent work. In the experiment, it was found that acupuncture can significantly stimulate brain activities in the frontal lobe (Figure 6). According to this phenomenon, it is necessary to appropriately increase the EEG recording density in this area in subsequent work. If necessary, consideration can also be given to using electrocorticography (ECoG) to observe intracranial EEG, spikes, etc.

The small sample size, which is 24, is another limitation of this work. This is also why we emphasize in the title that this is a pilot study. However, considering the experiment complexity and the current computational results, we still found some interesting phenomena in the existing dataset, e.g., VA can evoke similar EEG activities as in MA, and VA at ST36 shows significant activations in the frontal lobe. This will provide important insights for us and peer researchers in subsequent work. It should be emphasized that the experiments discussed in this paper are ongoing. We will continue to replicate relevant experiments and analyses on a larger sample size to make the results and experiments more robust.

### 5.5 Future work

This study provides a valuable reference for the use of VR scenarios for acupuncture stimulation. In addition to the work limitations as mentioned in the above subsection, the next steps of the research will unfold from multiple aspects. First, revealing the mechanisms underlying the generation of brain activities under VA stimulation is one of the research priorities. Second, conducting an in-depth analysis of the effects of VA regarding age and gender. Traditional acupuncture shows noticeable differences among subjects (Yang et al., 2020; Fan et al., 2021), especially when concerning age and gender. It's worth studying how VA performs across different age and gender groups. Finally, given the diversity of acupuncture points, analyzing the results and effects of VA stimulation at different points is also a crucial aspect of our future study. Through a series of studies, we hope to propose an objectively traceable, affordable, and efficient option for acupuncture. The new method aims to provide a theoretical and experimental basis for solutions in related medical, health, rehabilitation, and other related applications.

## 6 Conclusion

Our study combined VR-based acupuncture VA with MA and observed minimal EEG differences between the two, especially notable 24 h post-initial MA session. This research suggests potential gender variations in VA's efficacy, with females displaying less significant differences compared to MA. The experiment aimed to determine if virtual video stimulation in VA could significantly alter EEG activities compared to MA. Analysis of EEG data from subjects undergoing both treatments revealed only slight disparities between VA and MA, particularly a day after the first MA session. These initial findings indicate VR-based acupuncture's promise as an innovative method in both research and clinical settings, particularly within the realms of BCI technology and traditional acupuncture therapy. Future studies will delve deeper, employing a larger sample size and a broader array of acupuncture points. This approach is intended to enhance our comprehension of acupuncture's neural mechanisms and facilitate the integration of traditional practices with modern technological advancements. These expansions aim to provide a more thorough understanding of acupuncture from neural and brain-based perspectives, offering richer insights into the synergy between conventional acupuncture techniques and contemporary technological applications. Such research holds the potential to significantly impact BCI technology and acupuncture therapy.

## Data availability statement

The raw data supporting the conclusions of this article will be made available by the authors, without undue reservation.

## Ethics statement

The studies involving humans were approved by the Research Ethics Board of the Second Affiliated Hospital of Nanjing University of Chinese Medicine. The studies were conducted in accordance with the local legislation and institutional requirements. The participants provided their written informed consent to participate in this study.

## Author contributions

YZ: Writing – original draft, Methodology. WH: Methodology, Writing – review & editing. ZZ: Data curation, Writing – review & editing. HZ: Writing – review & editing, Data curation. JX: Data curation, Writing – review & editing. JZ: Writing – review & editing. DC: Writing – review & editing, Writing – original draft. JG: Writing – review & editing.
