# Supplementary material for: A novel acupuncture technique at the Zusanli point based on virtual reality and EEG: a pilot study
Source: Front Neurosci. 2024 May 9;18:1269903. doi: 10.3389/fnins.2024.1269903 (PMC11114168; doi:10.3389/fnins.2024.1269903)
Supplement: Supplementary file 1 [file Image_1.PDF]

# Supplementary Material

## 1 SUPPLEMENTARY FIGURES

The supplementary material primarily displays the topography for 24 subjects as shown in Figure S1 to S24, with the values in the graphs representing $_m$ under the condition that $f \in [1, 50]Hz$. The specific calculation method for $S_m$ is outlined in Equations S1 and S2.

Channel-weighted frequency power summation is a technique that emphasizes the significance of various frequency bands by multiplying the Power Spectral Density by its frequency and aggregating the results across channels, capturing both their importance and spatial distribution. The PSD for every channel in segment $\psi_n$ is calculated as:

$$p^m_{n,f} = \left| \frac{1}{Q} \sum_{k=0}^{Q-1} w(k)\psi^m_n(k)e^{-j2\pi fkT} \right|^2 \tag{S1}$$

where: $\psi^m_n(k)$ represents the $k$-th sample of the $m$-th channel in segment $t_n$, $Q$ is the length of the segment, $w(k)$ is the windowing function applied to the segment, $f \in [1, 50)$ is the frequency variable, $T$ is the sampling period. $p^m_{n,f}$ is the power at frequency $f$ for the segment $t_n$ of the $m$-th channel.

To investigate the specific impact locations of acupuncture, we multiply each segment, $t^m_n(k)$ obtained from Equation S1 by its corresponding frequency for each frequency $f$, within the PSD $p^m_{n,f}$, then average this across all segments within an event, as shown in the following equation:

$$S_m = \frac{1}{N} \sum_{n=1}^{N} \sum_{f \in F} f \times p^m_{n,f} \tag{S2}$$

where: $N$ is the total number of segments in $v$-th event, $F \in [1, 50)$ is the set of all frequencies for computed the PSD, $S_m$ represents the weighted spectral content of the $m$-th channel within the $v$-th event.

Each map corresponds to a distinct event and illustrates the weighted spectral content $S_m$, as computed by equation S2. The subplots are labeled MA1, MA2, VA1, VA2, VA3, VA4, showing power distribution across the scalp. Power levels are denoted by the color bar scale in decibels (dB). Vibrant central colors mark areas of high power, and the perimeter's cooler colors denote areas of low power.

### 1.1 Figures

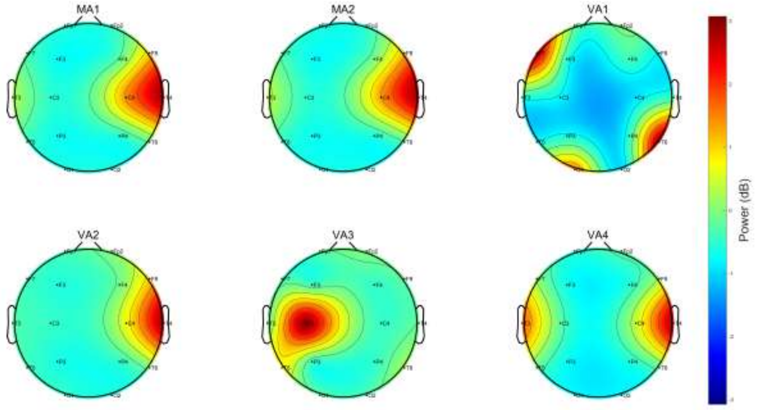


**Figure S1.** Topography of Sub01.

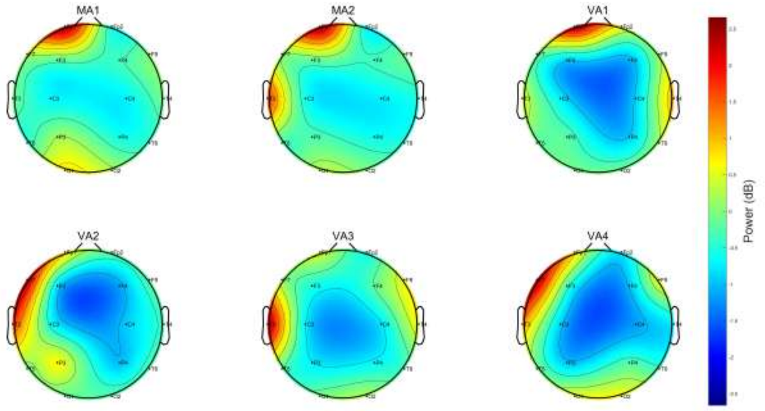


**Figure S2.** Topography of Sub02.

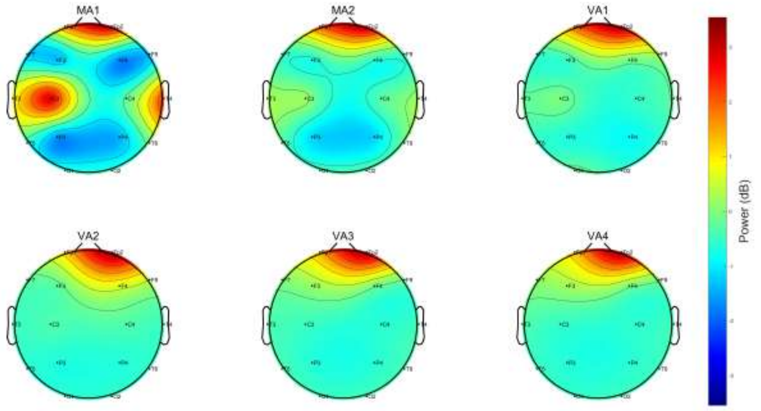


**Figure S3.** Topography of Sub03.

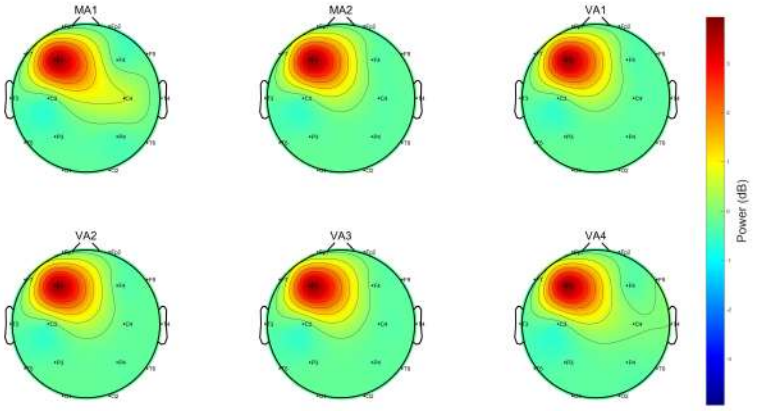


**Figure S4.** Topography of Sub04.

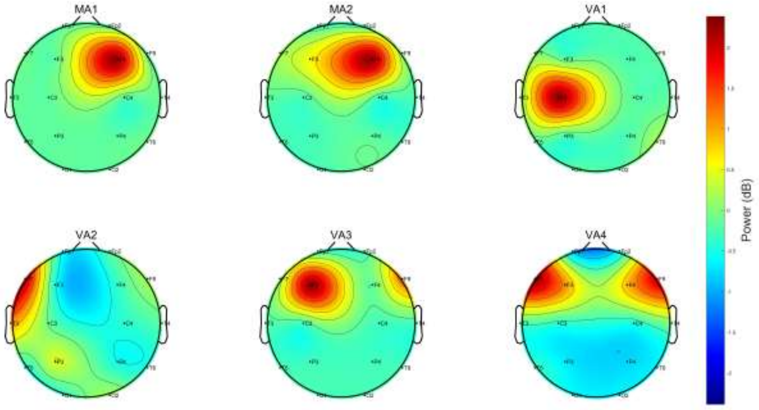


**Figure S5.** Topography of Sub05.

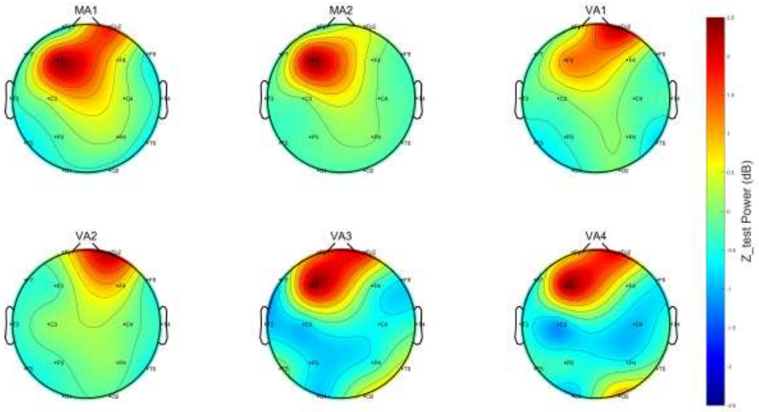


**Figure S6.** Topography of Sub06.

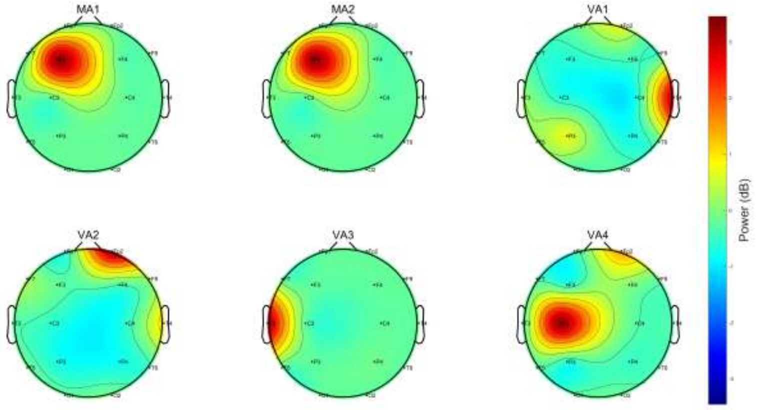


**Figure S7.** Topography of Sub07.

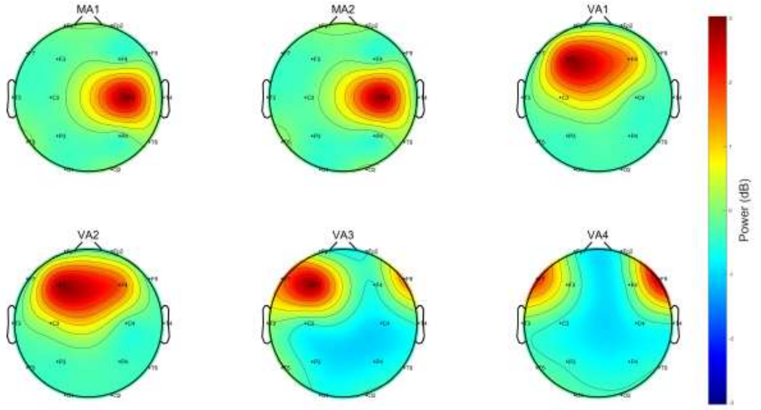


**Figure S8.** Topography of Sub08.

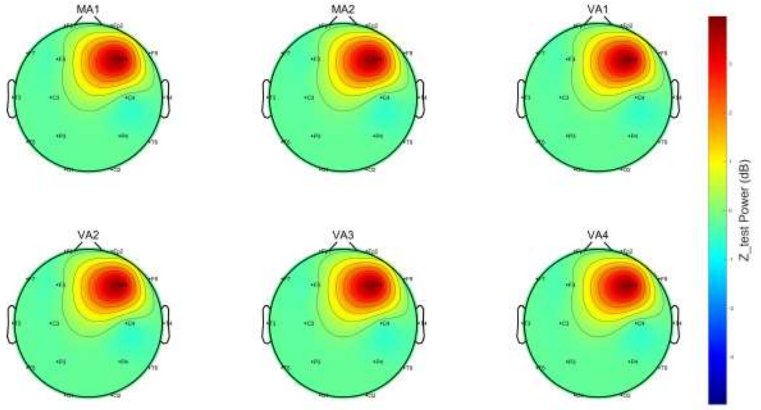


**Figure S9.** Topography of Sub09.

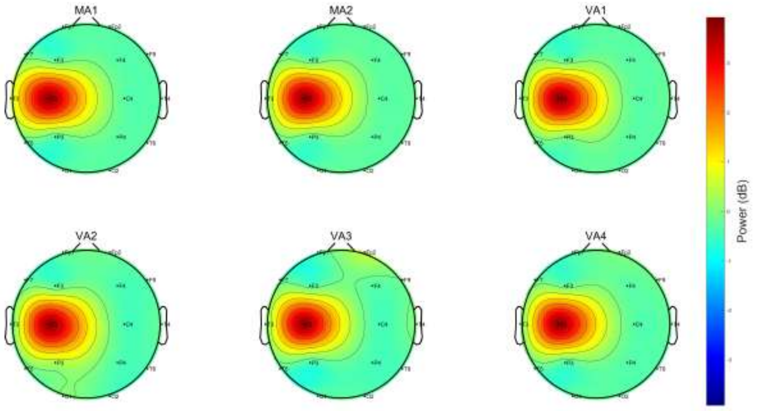


**Figure S10.** Topography of Sub10.

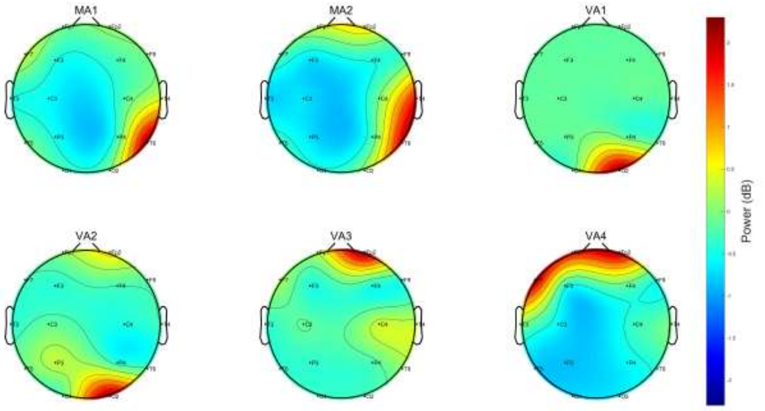


**Figure S11.** Topography of Sub11.

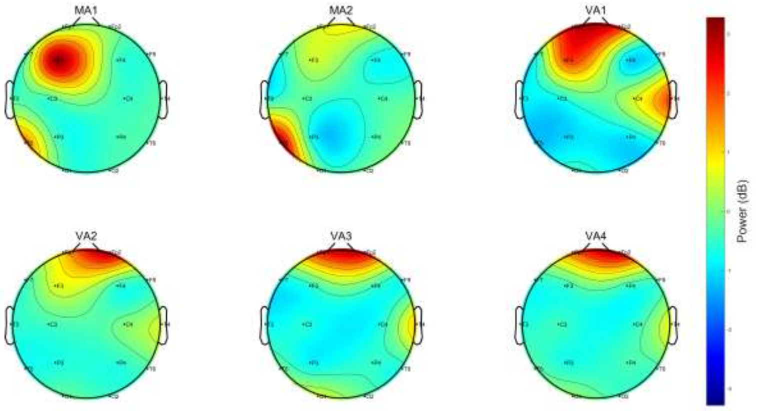


**Figure S12.** Topography of Sub12.

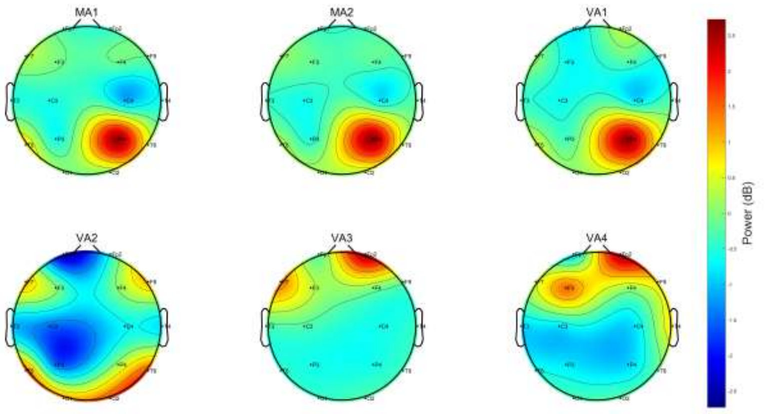


**Figure S13.** Topography of Sub13.

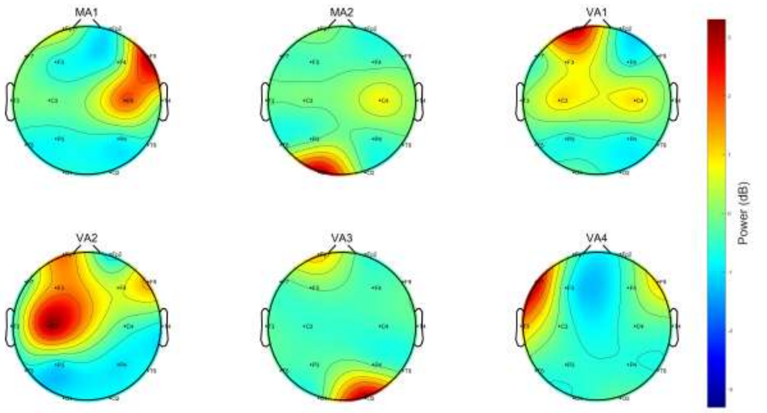


**Figure S14.** Topography of Sub14.

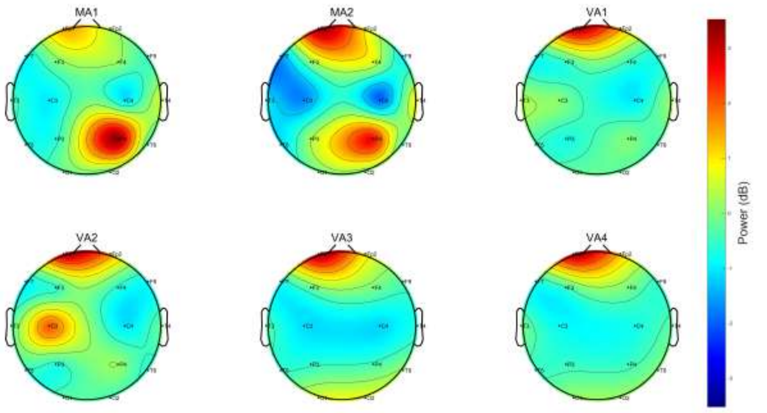


**Figure S15.** Topography of Sub15.

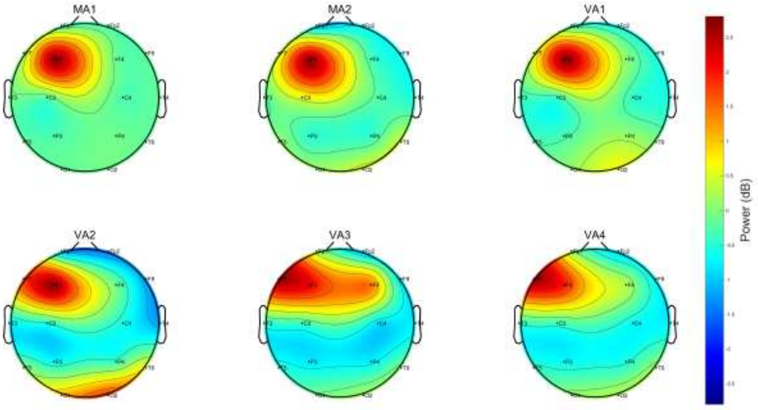


**Figure S16.** Topography of Sub16.

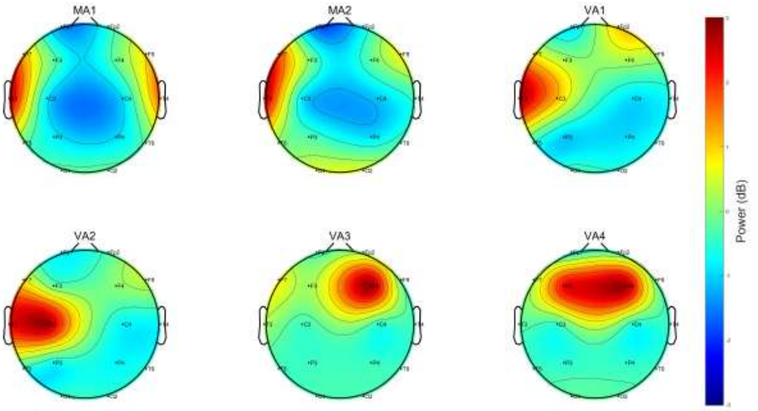


**Figure S17.** Topography of Sub17.

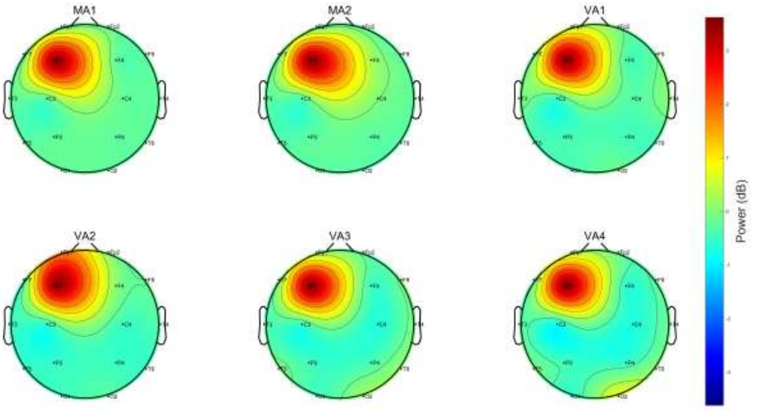


**Figure S18.** Topography of Sub18.

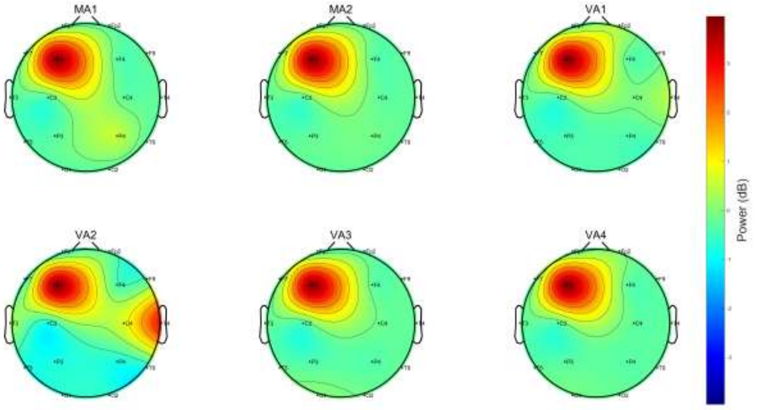


**Figure S19.** Topography of Sub19.

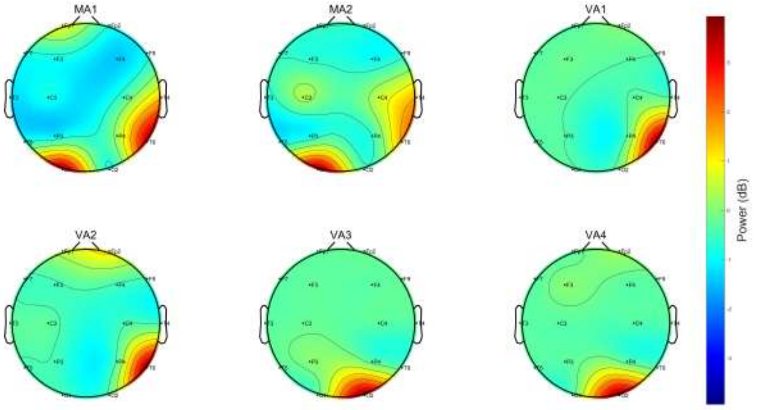


**Figure S20.** Topography of Sub20.

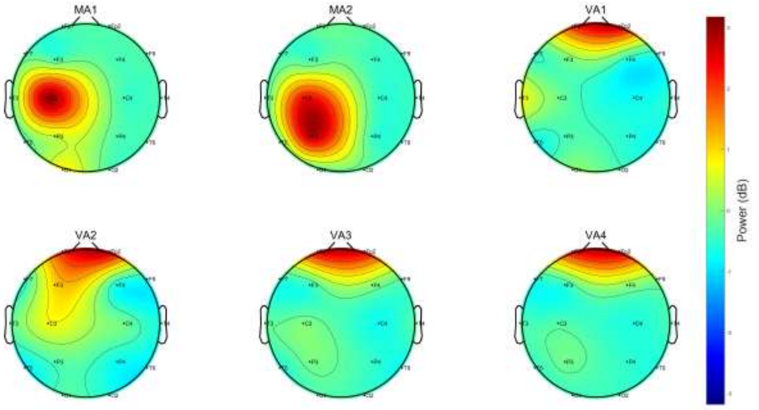


**Figure S21.** Topography of Sub21.

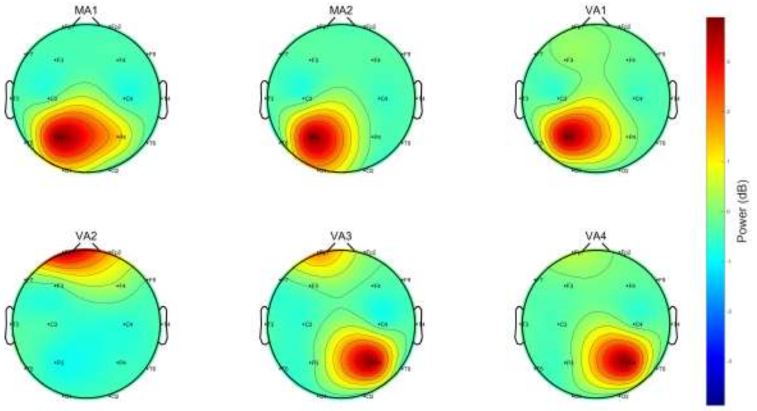


**Figure S22.** Topography of Sub22.

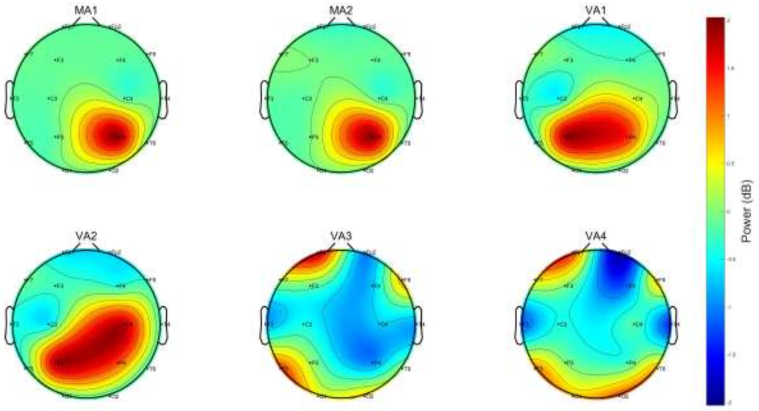


**Figure S23.** Topography of Sub23.

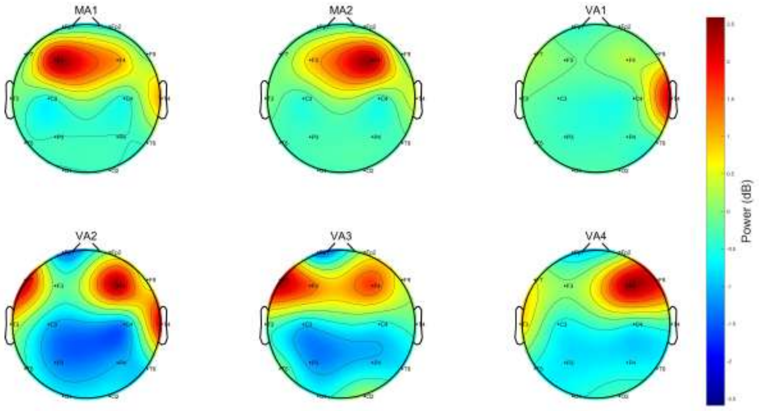


**Figure S24.** Topography of Sub24.
